# Feasibility, Usability and Acceptance of a Multi-Component Cognitive Intervention Using Immersive Virtual Reality and Telemedicine in Individuals with Subjective Cognitive Decline

**DOI:** 10.3390/jcm15051700

**Published:** 2026-02-24

**Authors:** Maria Stefania De Simone, Alberto Costa, Silvia Zabberoni, Gaetano Tieri

**Affiliations:** 1 Department of Economics, Psychology, Communication, Education, and Motor Sciences, Niccolò Cusano University, 00166 Rome, Italy; alberto.costa@unicusano.it (A.C.); silvia.zabberoni@unicusano.it (S.Z.); 2 Laboratory of Neuropsychology of Memory, Department of Clinical Neuroscience and Neurorehabilitation, IRCCS Santa Lucia Foundation, 00179 Rome, Italy; 3 Virtual Reality and Digital Neuroscience Lab, Department of Law and Digital Society, University of Rome Unitelma Sapienza, 00161 Rome, Italy; gaetano.tieri@unitelmasapienza.it

**Keywords:** subjective cognitive decline, cognitive training, immersive virtual reality, telemedicine, usability, feasibility

## Abstract

**Background/Objectives**: Dementia is a major global health challenge, with prevention strategies increasingly focusing on the preclinical stage of Subjective Cognitive Decline (SCD). This study aimed to evaluate the feasibility, usability, and acceptability of a 5-week immersive virtual reality and telemedicine-based multicomponent intervention, combining cognitive training with a health and lifestyle education program, in individuals with SCD. **Methods**: Thirty-nine individuals with SCD were randomly allocated to either the multi-component intervention (MC-I; *n* = 19) or the cognitive-only intervention (CO-I; *n* = 20). Both programs were delivered remotely via head-mounted displays and monitored through a telemedicine platform. Feasibility was assessed through retention, adherence, and safety measures. Post-intervention, participants completed the System Usability Scale (SUS), User Satisfaction Evaluation Questionnaire (USEQ), NASA Task Load Index (NASA-TLX), and Simulator Sickness Questionnaire (SSQ). **Results**: High feasibility was demonstrated by a 100% retention rate and 92% adherence. Both groups reported “excellent” usability (SUS mean: 84.04) and high satisfaction (USEQ mean: 26.7), with no significant differences between groups. The NASA-TLX reflected a moderate workload (mean: 56.1), characterized by high mental demand but low frustration. Safety was confirmed by remarkably low SSQ scores, indicating negligible cybersickness. **Conclusions**: The results provide strong preliminary evidence that a home-based, multi-component IVR intervention is safe, usable, and highly accepted by individuals with SCD. Integrating lifestyle education does not increase the perceived burden, supporting the scalability of this remote digital approach for dementia secondary prevention.

## 1. Introduction

Dementia represents one of the most significant health and societal challenges of our time, currently affecting over 55 million people worldwide—a number projected to double every 20 years due to global population aging [1]. Given the current lack of effective disease-modifying pharmacological treatments, research has increasingly shifted its focus toward prevention strategies [2]. The latest report from the Lancet Commission estimated that up to 45% of dementia cases could be prevented or delayed by addressing modifiable risk factors, including physical inactivity, low cognitive engagement, and poor dietary habits [3].

Alzheimer’s Disease (AD), the leading cause of dementia, is characterized by a long preclinical phase where pathophysiological changes begin decades before the onset of objective clinical symptoms [4]. Subjective Cognitive Decline (SCD)—defined as a self-perceived decline in cognitive capacity despite normal performance on standardized neuropsychological tests—is increasingly recognized as the earliest detectable stage of this continuum [5]. Individuals with SCD have a risk of progression to Mild Cognitive Impairment (MCI) or dementia up to five times higher than those without cognitive concerns [6,7]. This at-risk state offers a crucial “window of opportunity” for secondary prevention, as brain plasticity and functional independence are still largely preserved [8,9]. Thus, there is a compelling need for effective interventions that address the specific health needs of the SCD population, representing a critical step in secondary prevention and the slowing of cognitive decline.

Systematic reviews and meta-analyses suggest that non-pharmacological interventions, including those targeting cognitive training, exercise training, and health education, can have beneficial effects on various health dimensions of adults with SCD (i.e., objective and subjective cognitive functioning, physical health, quality of life, anxiety) and may postpone the manifestation of clinical symptoms [10,11,12,13]. For example, a recent systematic review and meta-analysis by Sheng and colleagues demonstrated that non-pharmacological interventions, particularly cognitive training and physical exercise, significantly improved global cognitive function and specific domains such as memory and executive functions in individuals with SCD [12]. Furthermore, a more recent network meta-analysis by Yu et al. provided a comprehensive comparison of different intervention types, highlighting that multi-component interventions—combining multiple strategies like cognitive training, physical activity, and dietary guidance—often outperform single-domain approaches [13]. Their findings suggest that these integrated programs are particularly effective in improving not only objective cognition but also psychological well-being and health-related quality of life.

However, despite these promising results, several gaps remain. Roheger et al. emphasized that while education programs and cognitive interventions show potential for improving memory performance, the evidence is still characterized by significant heterogeneity in study protocols and outcome measures [9]. This variability makes it difficult to establish a “gold standard” for SCD treatment. Moreover, as noted by Smart et al., many studies have been limited by small sample sizes and a lack of long-term follow-up, underscoring the need for more rigorous, large-scale randomized controlled trials to confirm the durability of these benefits and their potential to delay the transition to clinical stages of dementia [10].

Among non-pharmacological interventions, computerized cognitive training offers several advantages over traditional methods, providing tailored, adaptive, and domain-specific tasks [14]. In this context, Immersive Virtual Reality (IVR) further enhances these interventions by fostering neuroplasticity through multisensory stimulation and ecologically valid tasks, providing a high sense of presence that increases motivation [15,16,17]. Furthermore, integrating these technologies with telemedicine platforms allows for remote, home-based interventions, ensuring continuity of care and accessibility [18,19].

To date, the study by Kang and colleagues represents the only randomized controlled trial that evaluated the efficacy of fully IVR cognitive training in the SCD population, reporting significant improvements in visuospatial functions and functional connectivity [20]. Moreover, Arlati et al. reported high levels of acceptance and usability among older adults with both objective and subjective cognitive decline, emphasizing that immersive technology is well-tolerated when designed with user-centric features [21].

In parallel, the shift toward home-based rehabilitation has gained traction, as individuals with SCD often favor the flexibility and continuity of care it provides [22]. Recent studies on mobile-based multidomain programs and telehealth-mediated training [23,24] confirm that remote digital interventions are feasible if adequate technical support is provided to bridge the “digital divide”. Despite the growing interest in digital health, evidence regarding the efficacy, acceptability, and usability of a multi-component training delivered entirely through home-based IVR remains underexplored in the SCD literature.

To fill this gap, to our knowledge we developed the first fully immersive VR and telemedicine-based multi-component intervention specifically designed for individuals with SCD, combining cognitive training with a health and lifestyle education program for home-based delivery. The comprehensive methodology of this intervention, the efficacy of which is currently being evaluated through a randomized controlled trial, was detailed in our published study protocol [25]. In a preliminary pilot study, we evaluated the system usability and feasibility of a single in-presence session of cognitive training, lasting approximately 30 min and comprising two novel IVR memory tasks (i.e., the Virtual Face-Name Memory task and the Virtual Object-Location Memory task) in a sample of healthy young adults [26]. The results highlighted a positive user experience, minimal cybersickness, and low frustration ratings, suggesting that the procedure is a feasible and engaging tool for cognitive intervention.

While clinical efficacy outcomes are being addressed in our ongoing randomized controlled trial, the present study aimed to evaluate the feasibility, usability, and acceptability of the entire 5-week IVR and telemedicine-based multi-component intervention in a sample of individuals with SCD. Our goal was to examine the long-term sustainability of the training within a home-based setting and specifically within the target clinical population. For this purpose, we utilized a comprehensive set of standardized questionnaires to assess usability, perceived workload, and user satisfaction, as well as measures of retention, adherence and safety of the proposed intervention, comparing the multi-component approach (MC-I) with cognitive-only training (CO-I).

The remainder of this paper is organized as follows: Section 2 (Materials and Methods) details the participant recruitment process, the multi-component IVR intervention protocol, and the standardized measures used for assessment. Section 3 (Results) presents the findings regarding feasibility, system usability, user satisfaction, and perceived workload. Section 4 (Discussion) interprets these results in the context of existing literature and discusses the clinical implications and limitations of the study. Finally, Section 5 (Conclusions) summarizes the main findings and suggests future research directions.

## 2. Materials and Method

### 2.1. Participants

This study included a total of 59 consecutive individuals with Subjective Cognitive Decline (SCD) recruited from the Alzheimer’s Disease Unit of the Santa Lucia Foundation (Rome, Italy), who sought clinical attention due to self-reported cognitive complaints. All participants underwent a formal clinical and neuropsychological evaluation as part of the standard diagnostic process. Inclusion criteria were defined in accordance with current clinical guidelines for SCD and required: (a) age over 55 years; (b) absence of neurological or psychiatric disorders; (c) no history of alcohol or drug abuse; (d) a Mini-Mental State Examination (MMSE) score ≥28; (e) age-appropriate cognitive functioning, confirmed by performance above the normality cut-off scores on all tests within the extensive neuropsychological battery administered during the screening phase; and (f) subjective concern regarding their perceived cognitive decline [5,27].

All participants gave their written informed consent to take part in the study in accordance with the Declaration of Helsinki. The protocol utilized in this study was approved by the Territorial Ethics Committee of Lazio Area 5 (experiment register N.81/SL/23).

### 2.2. Procedure

Detailed information regarding the study design and procedures utilized in this double-blind, randomized controlled trial (RCT) is provided elsewhere [25]. Briefly, after the screening phase and the collection of baseline outcome measures, the 59 participants were randomly allocated to one of three experimental conditions (ratio 1:1:1) using a stratified randomization method based on age: (1) multi-component intervention (MC-I, *n* = 19), consisting of cognitive IVR training plus a health and lifestyle education program; (2) cognitive-only intervention (CO-I, *n* = 20), consisting of cognitive IVR training plus an active control for the education program; and (3) active control intervention (AC-I, *n* = 20), including active controls for both the cognitive training and the education program. To ensure allocation concealment, the randomization sequence was generated by an independent researcher not involved in the recruitment or assessment phases.

All interventions (i.e., MC-I, CO-I, AC-I) were implemented in an immersive virtual reality environment. Participants completed their respective intervention at home using standalone Meta Quest 2 (Meta Platforms Inc., Menlo Park, CA, USA) head-mounted displays (HMD). These HMDs, which feature a high-resolution LCD (1832 × 1920 pixels per eye) and a 72 Hz refresh rate to ensure high immersion and visual comfort, were provided to each participant at the end of an in-person onboarding session. The HMDs were remotely controlled and monitored in real-time via a dedicated telemedicine platform that allowed the research team to track session completion (e.g., accuracy, timing), manage scheduling, and provide technical support. For more information, please see De Simone et al. [25].

The MC-I condition comprised cognitive IVR training (3 sessions/week, about 30 min each) and a health and lifestyle education program (1 session/week, about 20 min each), for a total of 20 sessions over 5 consecutive weeks. The cognitive training included two tasks that target specific functions found to be early affected in SCD individuals [27,28]. First, the Virtual Face Name Memory training is a long-term associative memory task designed to train the ability to learn and recall name-face-occupation associations. This included immediate and 10-min delayed recall, in both cued-recall and recognition test formats. Second, the Virtual Object-Location Memory training is a visuospatial working memory binding task designed to train the ability to integrate and retain multiple features within an object and in relation to its spatial context.

The health and lifestyle education program consisted of five immersive 360° VR videos aimed at increasing awareness and knowledge of risk factors for cognitive decline and dementia (e.g., dietary habits, physical activity, cognitive engagement).

The CO-I condition included the same cognitive IVR training as the MC-I (3 sessions/week, about 30 min each) and an active control for the education program (1 session/week, about 20 min each). The latter consisted of five immersive 360° VR videos on non-health related topics (e.g., science and history) serving as a placebo-like condition for the educational component.

Finally, the AC-I condition included an active cognitive control, serving as a placebo for the experimental cognitive IVR training, and the same educational active control used in the CO-I group.

### 2.3. Measures

Upon completion of the 5-week IVR training, participants in the experimental arms (i.e., MC-I and CO-I) completed five standardized self-report questionnaires to evaluate the usability and acceptability of the intervention: the System Usability Scale (SUS, the User Satisfaction Evaluation Questionnaire (USEQ, the NASA Task Load Index (NASA-TLX), and the Simulator Sickness Questionnaire (SSQ) [29,30,31,32]. The AC-I group, serving as a placebo control for the clinical trial, was not included in this assessment as they were not exposed to the specific IVR cognitive training and educational modules under investigation; therefore, their feedback would not have been relevant for evaluating the system’s technical usability and workload. The assessment lasted approximately 15 min per participant and was conducted within two days of the intervention’s end. Given the 5-week duration of the protocol, this single post-intervention assessment was deemed appropriate to allow participants enough time to fully familiarize themselves with the immersive VR technology and the telemedicine platform. This ensured that the usability and acceptability ratings reflected a consolidated user experience, rather than being influenced by the initial learning curve or the novelty effect of the devices.

The SUS provided a subjective evaluation of the interface’s usability. This 10-item instrument quantifies both learnability and satisfaction (e.g., “I found the various functions in this system were well integrated”, “I think that I would like to use this system frequently”). Items are rated on a 5-point Likert scale, ranging from 1 (Strongly Disagree) to 5 (Strongly Agree), with a total score of 0 to 100. A SUS score above 68 is considered above-average usability, and a score above 80 is considered high usability [33,34].

The USEQ was used to assess users’ satisfaction with the IVR system. It comprises six items (e.g., “Did you enjoy your experience with the system?”, “Was the system easy to use?”) rated on a 5-point Likert scale ranging from 1 to 5. Each item evaluates a specific dimension, including self-perceived satisfaction (“Experience enjoyment”), efficacy (“Successful use”), efficiency (“Ability to control”), ease of use (“Clarity of information”), fatigue (“Discomfort”), and the utility of the performed exercise (“Perceived utility”). The total score ranges from 6 (poor satisfaction) to 30 (excellent satisfaction).

The NASA-TLX measured perceived workload across six dimensions: mental demand, physical demand, temporal demand, effort, performance, and frustration/stress level. It comprised six items (e.g., “How mentally demanding was the task?”, “How hard did you have to work to accomplish your level of performance?”), rated using a numerical visual scale ranging from zero to 100. Scoring ranges 0 to 100, with higher values reflecting a greater perceived workload. Based on established benchmarks, scores exceeding 60 are indicative of overstraining, while scores below 37 suggest understraining [35].

The SSQ was administered to assess users’ level of sickness following IVR exposure, based on subjective ratings of the severity of 16 symptoms, including general discomfort, fatigue, headache, nausea, and vertigo. Each of the16 items is rated on a 4-point scale, ranging from 1 (absent) to 4 (severe). The ratings for individual symptoms are divided into three non-exclusive sub-scales that represent symptoms of nausea (N), oculomotor disturbance (O), and disorientation (D). The total score results from the sum of the scores of the three subscales and ranges from 0 to 48. A score below 10 is traditionally considered to be normal, significant symptoms are indicated by scores between 10 and 15, while scores over 20 indicate potential issues with simulator tolerance [36].

Feasibility was assessed in terms of retention, compliance, and safety of the proposed experimental intervention. Retention was recorded as the number (proportion) of participants who completed the 5-week intervention. Compliance was determined by the number (proportion) of training sessions per week and the overall adherence to the prescribed program. Safety was determined based on the incidence of adverse events (e.g., dizziness or discomfort) that led to discontinuation or modification of the intervention.

### 2.4. Data Analysis

Statistical analyses were performed using IBM SPSS Statistics (Version 27.0; IBM Corp., Armonk, NY, USA). Demographic and clinical characteristics were summarized using descriptive statistics, with con-tinuous variables expressed as mean ± standard deviation (SD) and categorical variables as frequencies and percentages (%). Data normality was assessed using the Shapiro-Wilk test. To ensure baseline com-parability between the two experimental groups (CO-I vs. MC-I), independent samples *t*-tests or Mann-Whitney U tests were used for continuous variables, while chi-squared (χ^2^) tests were applied to categorical variables. For post-training questionnaires (SUS, USEQ, NASA-TLX, and SSQ), descriptive statistics including means, SD, medians, and interquartile ranges (IQR) were utilized as appropriate. Differences in questionnaire scores between groups were assessed using independent samples *t*-tests for normally distributed data (NASA-TLX) and non-parametric Mann-Whitney U tests for variables that deviated from normality (SUS, USEQ, and SSQ). For all analyses, statistical significance was set at *p* < 0.05.

## 3. Results

### 3.1. Demographic and Clinical Data

Post-training questionnaires were collected from all 39 participants allocated to the experimental groups (CO-I: *n* = 20; MC-I: *n* = 19). The total sample had a mean age of 66 ± 6.3 years, an average education of 14.1 ± 3.2 years, and a mean MMSE score of 29.1 ± 1.0. The sex distribution was 8 males and 31 females. No significant differences were found between the CO-I and MC-I groups regarding demographic data—including age (CO-I: 66 ± 6.3; MC-I: MC-I: 66 ± 6.3; t(37) = 0.02, *p* = 0.98), education (CO-I: 14.1 ± 3.0; MC-I: 14.1 ± 3.4; z = 0.13, *p* = 0.90), and sex (CO-I: 4 males/16 females; MC-I: 4 males/15 females; χ^2^(1) = 0.01, *p* = 0.94)—or MMSE scores (CO-I: 29.3 ± 0.98; MC-I: 28.9 ± 0.97; z = 1.26, *p* = 0.21), confirming baseline comparability between the two experimental arms.

### 3.2. Feasibility

The intervention demonstrated high feasibility. All 39 participants completed the study with no dropouts, resulting in a retention rate of 100%. At the 5-week mark, the adherence rate was 92%, with 36 out of 39 participants completing all 20 training sessions. The remaining three participants completed at least 90% of the prescribed sessions (18 out of 20 sessions). No serious adverse events were recorded during the study period.

### 3.3. Usability

**Figure 1:**
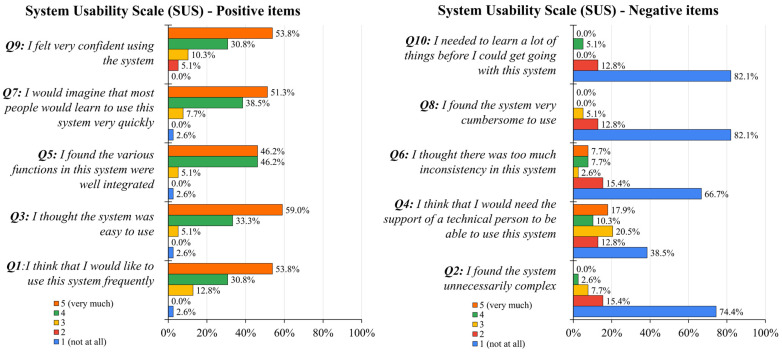
Subjective usability evaluation via the System Usability Scale (SUS). The bar chart illustrates the percentage distribution of participant responses on the 5-point Likert scale for each of the 10 items of the SUS.

System Usability Scale (SUS). Figure 1 illustrates the distribution of scores (in percentage) on the 5-point Likert scale for each item of the SUS questionnaire. Positive items (odd numbers) showed a high concentration of scores at the upper end of the scale (4 and 5), while negative items (even numbers) showed a predominant concentration at the lower end (1 and 2), collectively indicating high system usability and user satisfaction. The overall mean score was 84.04 (±11.8), which is indicative of excellent usability according to standard benchmarks. Specifically, 75% of the participants (*n* = 29) provided ratings of 80 or higher, and 15% (*n* = 6) scored between 68 and 79, indicating an acceptable-to-good level of usability [33,34]. No significant difference was found in SUS mean scores between the CO-I (83.5 ± 12.6) and MC-I groups (84.6 ± 11.2) (z = 0.21, *p* = 0.83).

On the 5-point Likert scale, scores exceeded a mean of 4 for items investigating satisfaction (Q1: “I think that I would like to use this system frequently”, mean score: 4.33 ± 0.9), ease of use (Q3: “I thought the system was easy to use”, mean score: 4.46 ± 0.8; Q7: “I would imagine that most people would learn to use this system very quickly”, mean 4.36 ± 0.8), efficiency of integration (e.g., Q5: “I found the various functions in this system were well integrated”, mean score: 4.33 ± 0.8), and confidence (e.g., Q9: “I felt very confident using the system”, mean score: 4.33 ± 0.9). These results suggest strong acceptance and high adoption potential for the IVR intervention.

**Figure 2:**
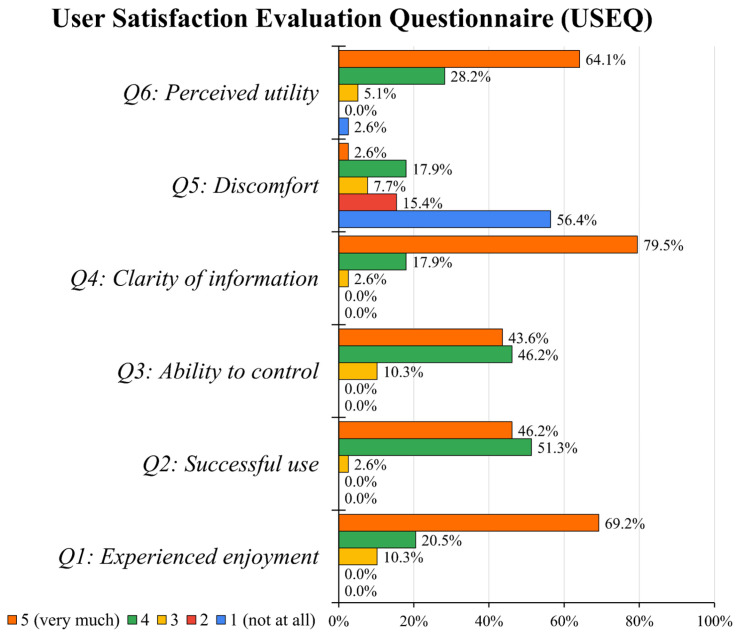
User satisfaction and system perception via the User Satisfaction Evaluation Questionnaire (USEQ). The bar chart displays the percentage of participant responses across the six USEQ items.

User Satisfaction Evaluation Questionnaire (USEQ). Figure 2 illustrates the distribution of scores on the 5-point Likert scale for each item of the USEQ questionnaire. High satisfaction is evidenced by the predominance of scores 4 and 5 across all positive items (1–4, 6) and by the predominance of scores 1 and 2 for the negative item (5). The overall mean score was 26.7 ± 2.8 (out of a maximum of 30), reflecting very high user satisfaction. The average item score was 4.4 ± 0.7. No significant differences were observed between the CO-I and MC-I groups in either the overall mean score (26.8 ± 2.9 vs. 26.6 ± 2.7; z = 0.29, *p* = 0.77) or the average item score (4.5 ± 0.7 vs. 4.4 ± 0.7; z = 0.11, *p* = 0.89).

On the 5-point Likert scale, mean scores exceeded 4 for all dimensions, including experience enjoyment (mean score 4.6 ± 0.7), successful use (mean score 4.4 ± 0.6), ability to control (mean score 4.3 ± 0.7), clarity of information (mean score 4.8 ± 0.5), discomfort (reversed mean score 4.1 ± 1.3), and perceived utility (mean score 4.5 ± 0.8).

NASA Task Load Index (NASA-TLX). The scores for each NASA-TLX domain are summarised in Table 1. The overall mean workload score was 56.1 (±23.6), reflecting a moderate workload according to established benchmarks [35]. No significant difference in the total workload was found between the CO-I and MC-I groups (55.5 ± 24.5 vs. 56.7 ± 22.5; t(37) = 0.24, *p* = 0.81). The highest workload levels were reported in the mental demand (mean score 78.2 ± 17.2) and effort (mean score 68.7 ± 19.6) dimensions. Participants rated their performance (mean score 60.3 ± 24.5) and frustration (mean score 49.0 ± 25.2) at a moderate level. Conversely, physical demand (mean score 39.5 ± 27.3) and temporal demand (40.8 ± 27.2) were rated the lowest, suggesting that the IVR training was cognitively demanding and stimulating but physically comfortable, without imposing excessive time pressure.

Simulator Sickness Questionnaire (SSQ). The SSQ results are summarized in Table 2. Notably, 95% of participants (*n* = 37) reported total scores ranging from 0 to 10, which fall within the category of “no significant level of sickness” [36]. The mean total score was 4.54 ± 3.6 (median: 4, IQR: 4.5), reflecting the absence of—or only negligible—negative symptoms related to IVR usage. No significant differences were found in total scores between the CO-I and MC-I groups (4.80 ± 3.6 vs. 4.26 ± 3.7; z = 0.48, *p* = 0.63). Regarding the sub-scales of the SSQ, the mean scores were as follows: Nausea: 0.82 ± 1.1; Oculomotor: 2.38 ± 2.0; Disorientation: 1.33 ± 1.2. These findings confirm the safety and high tolerability of the IVR training protocol, with negligible side effects reported by the participants.

## 4. Discussion

The present study aimed to evaluate the feasibility, usability, and acceptability of a 5-week IVR- and telemedicine-based multi-component intervention, combining cognitive training and a health and lifestyle education program, specifically developed for individuals with SCD. For this purpose, a sample of 39 individuals with SCD, who were randomly allocated to either the multi-component intervention (MC-I, *n* = 19) or the cognitive-only intervention (CO-I; *n* = 20) completed a comprehensive set of questionnaires at the end of the 5-week intervention period, in order to evaluate usability, acceptability, and user satisfaction. Moreover, retention, adherence rates, and safety were assessed as primary indicators of the overall feasibility of the protocol. To our knowledge, this is the first study to explore the long-term sustainability of a multi-component intervention using IVR and delivered remotely in the home environment of individuals at risk for AD.

Our findings suggest high feasibility, as evidenced by a 100% retention rate with no dropouts recorded during the 5-week period. This is particularly noteworthy given that remote interventions often suffer from lower engagement and lack of individualized attention and support compared to in-person sessions [37], which in turn may result in reduced extrinsic motivation and lower adherence [38,39]. Notably, 92% of the participants demonstrated full compliance with the study protocol. Such high adherence suggests that the user-centric design of our IVR intervention, combined with a structured telemedicine support system, effectively mitigated the typical barriers associated with home-based training motivation [39]. At the same time, the remote administration of the intervention potentially favoured the integration of the training sessions into daily routines, mitigating constraints typical of traditional in-person interventions—such as time, resources, and transportation—while granting participants greater flexibility and a sense of autonomy over their training schedule. This finding is consistent with the literature documenting the feasibility of remote intervention and IVR cognitive training in individuals with SCD [21,23,24,40].

Regarding safety, no significant adverse events were registered during the 5-week period. This was further corroborated by the SSQ (Simulator Sickness Questionnaire) scores, which were remarkably low in both the MC-I and CO-I groups. The absence of significant nausea, oculomotor strain, or disorientation indicates that the IVR tasks were safe and well-tolerated by older adults with SCD, even in a self-administered home setting. These results support the preliminary findings of our pilot study [26] and align with previous research indicating that immersive VR is a viable tool for older adults when appropriately designed [21].

Regarding system usability, the SUS (System Usability Scale) scores for both experimental groups (MC-I: 84.87 ± 8.16; CO-I: 82.25 ± 10.87) were well above the industry benchmark of 68, falling into the “excellent” category according to conventional norms [33,41]. Interestingly, the addition of the lifestyle education component in the MC-I group did not negatively impact the perceived ease of use. This points toward the possibility that multi-domain protocols, while inherently more complex in content, may be as accessible as single-domain interventions if the digital interface is designed to be intuitive and user-friendly. These findings are particularly relevant to the literature on computerized and IVR training in older populations, where technical complexity can potentially overshadow clinical benefits [42].

Importantly, these high usability ratings were complemented by the results of the USEQ (User Satisfaction Evaluation Questionnaire), where both groups achieved nearly 90% of the maximum possible score. The high USEQ scores across both groups indicate that the interface was intuitive enough to allow participants to focus on the cognitive tasks rather than the technology itself, effectively bridging the “digital divide” often cited as a barrier for older adults [23]. In particular, participants reported high levels of satisfaction regarding the clarity of information and the overall enjoyment and perceived utility of the training. Such high levels of engagement are crucial, as “enjoyment” is a known predictor of long-term adherence in interventional programs [24].

Regarding the perceived workload, the NASA Task Load Index (NASA-TLX) results provide a nuanced understanding of the cognitive effort required by the intervention. Both experimental groups (MC-I and CO-I) reported total workload scores that fall well within the optimal range [35], effectively avoiding the “overstraining” (>60) and “understraining” (<37) thresholds, which are typically associated with decreased performance, frustration, and eventual abandonment of the training [35]. A detailed analysis of the NASA-TLX subscales reveals a specific and desirable workload profile. Participants reported high scores in Mental Demand, indicating that the IVR memory tasks were perceived as cognitively challenging. In the context of cognitive rehabilitation, mental workload is considered a key driver for stimulating neuroplasticity and cognitive reserve [43,44]. Crucially, this high mental engagement was contrasted by low scores in the Frustration and Temporal Demand subscales. Furthermore, the high ratings in Performance (indicating that participants felt successful in accomplishing the tasks) combined with low frustration suggest a balanced challenge that is vital for older adults, as excessive frustration in digital interventions can reinforce technology-related anxiety and lower self-efficacy [23].

Despite the promising findings, this study has some limitations that should be acknowledged. First, the sample size is relatively small, and the predominantly female composition may limit the generalizability of the results to the broader SCD population. However, it should be noted that our study employed a consecutive recruitment pattern, which inherently reflects the demographic profile of the population seeking consultation for SCD at specialized center. This distribution is indeed consistent with the higher prevalence of SCD and memory concerns reported among women in the literature, as well as their higher clinical consultation rates [45,46]. Second, the five-week intervention duration is relatively short, and the lack of a long-term follow-up prevents us from determining the stability of the observed feasibility and acceptability over time. Furthermore, the assessment relied primarily on self-report measures, which may be subject to social desirability bias. Future large-scale randomized controlled trials with longer follow-up periods and a more balanced gender distribution are needed to confirm these preliminary findings and evaluate clinical efficacy.

## 5. Conclusions

In conclusion, our study provides promising preliminary evidence that a multi-component, home-based IVR intervention is feasible, usable, and highly accepted by individuals with SCD. These findings primarily support the practical implementation feasibility and scalability of the protocol, demonstrating that the integration of cognitive training and lifestyle education via telemedicine appears not to increase the perceived burden on the user. By establishing this foundational sustainability, the present work identifies a potentially scalable strategy for dementia prevention. Subsequent studies will investigate the clinical efficacy of this protocol across different health dimensions, including objective and subjective cognitive function, psychiatric symptoms, quality of life, and brain connectivity, with the aim of proposing clinical practice guidelines based on evidence from our ongoing randomized controlled trial [25].

## Figures and Tables

**Table 1:** Distribution of NASA-TLX dimension scores across the study sample. The table presents the frequency (*n*) and percentage (%) of participants falling into different workload intensity categories (from “Low” to “Very High”) for each of the six NASA-TLX dimensions.

NASA Score		Mental Demand		Physical Demand		Temporal Demand		Effort		Performance		Frustration	
		*n*	*%*	*n*	*%*	*n*	*%*	*n*	*%*	*n*	*%*	*n*	*%*
0–19	Low	0	0.0%	11	28.2%	9	23.1%	0	0.0%	3	7.7%	5	12.8%
20–39	Medium	2	5.1%	10	25.6%	11	28.2%	3	7.7%	5	12.8%	8	20.5%
40–59	Somewhat high	0	0.0%	7	17.9%	6	15.4%	7	17.9%	5	12.8%	9	23.1%
60–79	High	10	25.6%	3	7.7%	6	15.4%	14	35.9%	15	38.5%	11	28.2%
80–100	Very high	27	69.2%	8	20.5%	7	17.9%	15	38.5%	11	28.2%	6	15.4%

**Table 2:** Distribution of SSQ total scores and symptom categorization. The table presents the frequency (*n*) and percentage (%) of participants categorized by the severity of their negative symptoms following the IVR intervention according to SSQ total score.

SSQ Total Score	Categorisation	Number of Responses (%)	
0	No symptoms	6	(15.4%)
1–5	Negligible symptoms	20	(51.3%)
6–10	Minimal symptoms	11	(28.2%)
11–15	Significant symptoms	1	(2.6%)
15–20	Symptoms area concern	1	(2.6%)
>20	A bad intervention	0	(0%)

## Data Availability

The data presented in this study are available on request from the corresponding author due to privacy, legal, and ethical reasons.

## Abbreviations

AC-I	Active Control Intervention
AD	Alzheimer’s Disease
CO-I	Cognitive-Only Intervention
HMD	Head-Mounted Display
IQR	Interquartile Range
IVR	Immersive Virtual Reality
MC-I	Multi-Component Intervention
MCI	Mild Cognitive Impairment
MMSE	Mini-Mental State Examination
NASA-TLX	NASA Task Load Index
RCT	Randomized Controlled Trial
SCD	Subjective Cognitive Decline
SD	Standard Deviation
SSQ	Simulator Sickness Questionnaire
SUS	System Usability Scale
USEQ	User Satisfaction Evaluation Questionnaire
